# Bromelain and Curcumin Oral Supplementation for Refractory Inherited Retinal Dystrophy-Related Macular Oedema: Changes in Macular Thickness and Visual Acuity over 12 Months

**DOI:** 10.3390/ph19040602

**Published:** 2026-04-09

**Authors:** Mattia D’Andrea, Carmen Dell’Aquila, Lucilla Barbano, Feliciana Menna, Antonio Di Renzo, Gaspare Colacino, Marco Marenco, Roberto Dell’Omo, Vincenzo Parisi, Lucia Ziccardi

**Affiliations:** 1 Department of Sense Organs, Faculty of Medicine and Dentistry, Sapienza University of Rome, Viale del Policlinico 155, 00161 Rome, Italy; mattia.dandrea@uniroma1.it (M.D.); marco.marenco@uniroma1.it (M.M.); 2 IRCCS-Fondazione Bietti, Via Livenza 3, 00198 Rome, Italy; lucilla.barbano@fondazionebietti.it (L.B.); antonio.direnzo@fondazionebietti.it (A.D.R.); vincenzo.parisi@fondazionebietti.it or vincenzo.parisi@unicamillus.org (V.P.); lucia.ziccardi@fondazionebietti.it or lucia.ziccardi@unimol.it (L.Z.); 3 Department of Medical-Surgical Sciences and Biotechnologies, U.O.C. Ophthalmology, Sapienza University of Rome, Via Firenze 1, 04019 Terracina, Italy; feliciana.menna@gmail.com; 4 UOC Oftalmologia-San Giovanni Evangelista Hospital Tivoli, Via Antonio Parrozzani 3, 00019 Tivoli, Italy; colacinogaspare@gmail.com; 5 Department of Medicine and Health Sciences “V. Tiberio”, University of Molise, Via F. De Sanctis 1, 86100 Campobasso, Italy; roberto.dellomo@unimol.it; 6 Departmental Faculty of Medicine, UniCamillus-Saint Camillus International University of Health Sciences, 00131 Rome, Italy

**Keywords:** retina, inherited retinal dystrophies, retinitis pigmentosa, oedema, macula, bromelain, curcumin, nutraceutical, antioxidant

## Abstract

**Objectives**: To evaluate the long-term effects on retinal structure and visual function of oral bromelain and curcumin supplementation in patients with inherited retinal dystrophies (IRD) complicated by persistent cystoid macular oedema (CMO). **Methods**: We retrospectively studied 20 eyes with genetically confirmed IRD complicated by CMO, with refractory to systemic or local treatments performed for 6 months. We collected baseline (V1) and follow-up (V2) data from these IRD-CMO patients, who were continuously supplemented with oral bromelain and curcumin for 12 months. Outcome measures were the Snellen best-corrected visual acuity (BCVA) and central macular thickness (CMT) values, collected by spectral-domain optical coherence tomography (OCT). Based on OCT scans, we classified IRD-CMO as microcystic or macrocystic, performing this sub-grouping in two eye cohorts (*n* = 10). Baseline median BCVA and CMT differences in both groups were verified (Mann–Whitney test). For both CMO groups, changes from V1 to V2 in median BCVA and CMT values were evaluated (Friedman test). **Results**: At baseline, both the median BCVA and CMT values were significantly different in both groups (*p* < 0.01 and *p* < 0.001). Between V1 and V2, in the microcystic CMO group, a slightly improved median BCVA was found, whereas the median CMT was reduced; however, this did not reach statistical significance (*p* = 0.6 and *p* = 0.2, respectively). In the macrocystic CMO group, a significant stable median BCVA was found from V1 to V2, with concomitant significant reduction in median CMT (*p* < 0.05 for both comparisons). **Conclusions**: Retinal structural improvement and visual function preservation were observed after oral bromelain and curcumin supplementation in macrocystic IRD-CMO. It is likely that the vasogenic component in macrocystic CMO is more responsive to nutraceutical molecules than the degenerative microcystic component.

## 1. Introduction

Inherited retinal dystrophies (IRDs) represent a broad and clinically heterogeneous group of degenerative retinal disorders driven by pathogenic mutations in over 300 different genes [1].

The clinical course of IRD is characterised by gradual degeneration of photoreceptors (rod or cones), leading to structural damage, progressive visual field deficit and, as the condition advances, visual acuity decline related to central retinal involvement [1].

The most frequent phenotype, retinitis pigmentosa (RP), is mainly characterized by peripheral retinal degeneration with gradual constriction of the visual field and hemeralopia [2]. A total of 20% of RP cases follow an autosomal recessive inheritance pattern, while 25% are autosomal dominant, about 15% are X-linked, and 30% are classified as sporadic. The remaining 10% is constituted by rarer forms such as digenic and mitochondrial ones. RP can be isolated or part of multisystemic syndromes, such as Usher syndrome or Bardet–Biedl syndrome, accounting for 20–30% of cases [2,3].

In both syndromic or non-syndromic RP, macular pathology is often detected in advanced stages due to the centripetal spreading of retinal degeneration to the macula (macular atrophy) or in association with disease-related complications, such as vitreomacular traction or cystoid macular oedema (CMO) [4,5,6].

In 10–50% of RP patients, CMO complicates the phenotype and is mainly due to blood–retinal barrier (BRB) breakdown secondary to RPE and/or endothelial damage or dysfunction [6]. Because of BRB damage and activation of the inflammatory cascade at the level of the retina, proinflammatory substances like vascular endothelial growth factor (VEGF), adenosine, prostaglandins, histamine, insulin-like growth factor 1, tumour necrosis factor α (TNF-α), and interleukin-1α and interleukin-1β are released [7,8,9].

The gold-standard technique for detecting CMO in clinical ophthalmological settings is optical coherence tomography (OCT). It provides high-resolution cross-sectional imaging of retinal layers, enabling visualisation of even minute intraretinal fluid cysts [10,11]. In addition, OCT acquisition allows reproducible and non-invasive quantification of retinal thickness, facilitating accurate evaluation of central macular thickness (CMT) changes during treatment follow-up [12].

Therapeutic management of IRD-related CMO is crucial, especially in syndromic or non-syndromic RP patients suffering from severe impairment of the peripheral visual field. Indeed, prolonged macular involvement may further worsen the central vision and consequently patients’ visual prognosis over time. However, non-univocal findings of visual acuity deterioration have been reported, since concomitant disruption of the ellipsoid zone (EZ)’s integrity [12,13] may also affect visual acuity in IRD-CMO eyes.

The pathogenesis of IRD-related CMO is variable, swinging from tractional to vasogenic and degenerative etiologies. For this reason, it is challenging to establish an optimal therapeutic strategy, and treatment responses may vary [14]. Systemically administered carbonic anhydrase inhibitors (CAIs) are generally considered the first-line therapy [14], and in refractory cases, intravitreal administration of anti-VEGF agents or an intravitreal dexamethasone implant (IVT-DEX) may be employed [15].

Although these options can be used as a long-range treatment and can be repeated upon CMO recurrence, careful surveillance and management of systemic and ocular treatment-related complications, such as renal failure, electrolyte imbalances on one side, and intraocular pressure (IOP) elevation and cataract progression on the other side, are warranted [14,15]. Furthermore, the above-mentioned therapies may lead to an incomplete resolution of CMO, and, in some cases, are not tolerated or sustainable in the long term.

Some nutraceutical molecules have been revealed to be effective in ameliorating oedema of vasogenic or inflammatory origins [16,17,18], such as diabetic macular oedema (DMO) [19], or as a supplementary treatment to prolong/stabilise the effect of anti-VEGF therapy [20].

The aim of this study is to evaluate the anti-inflammatory and antioxidant effects of bromelain and curcumin on eyes affected by refractory RP-related CMO over 12 months of treatment.

## 2. Results

A total of 20 eyes from 14 patients with IRD-related CMO in one eye and 3 patients with IRD-related CMO in both eyes were included in the study. Of the 14 patients with monolateral IRD-related CMO, seven eyes from 7 patients were microcystic and seven eyes from 7 patients had macrocystic CMO. The three patients with bilateral IRD-related CMO provided six eyes: three eyes with microcystic and three eyes with macrocystic CMO. This condition was evident at V1 and V2; therefore, those 6 eyes were considered independent data points. CMO was related to RP in 12 eyes and to Usher Syndrome type II in 8 eyes.

The mean age ± 1 standard deviation (SD) at the time of V1 evaluation was 43 years. A total of 16 eyes had been previously treated with topical and/or systemic acetazolamide therapy for more than 6 months without reduction in the CMT or with recurrent oedema during treatment, and 4 eyes had been previously treated with repeated IVT-DEX. Among these, CMO recurred in two cases, while the remaining two developed treatment-related complications that ultimately required discontinuation of therapy.

**Figure 1:**
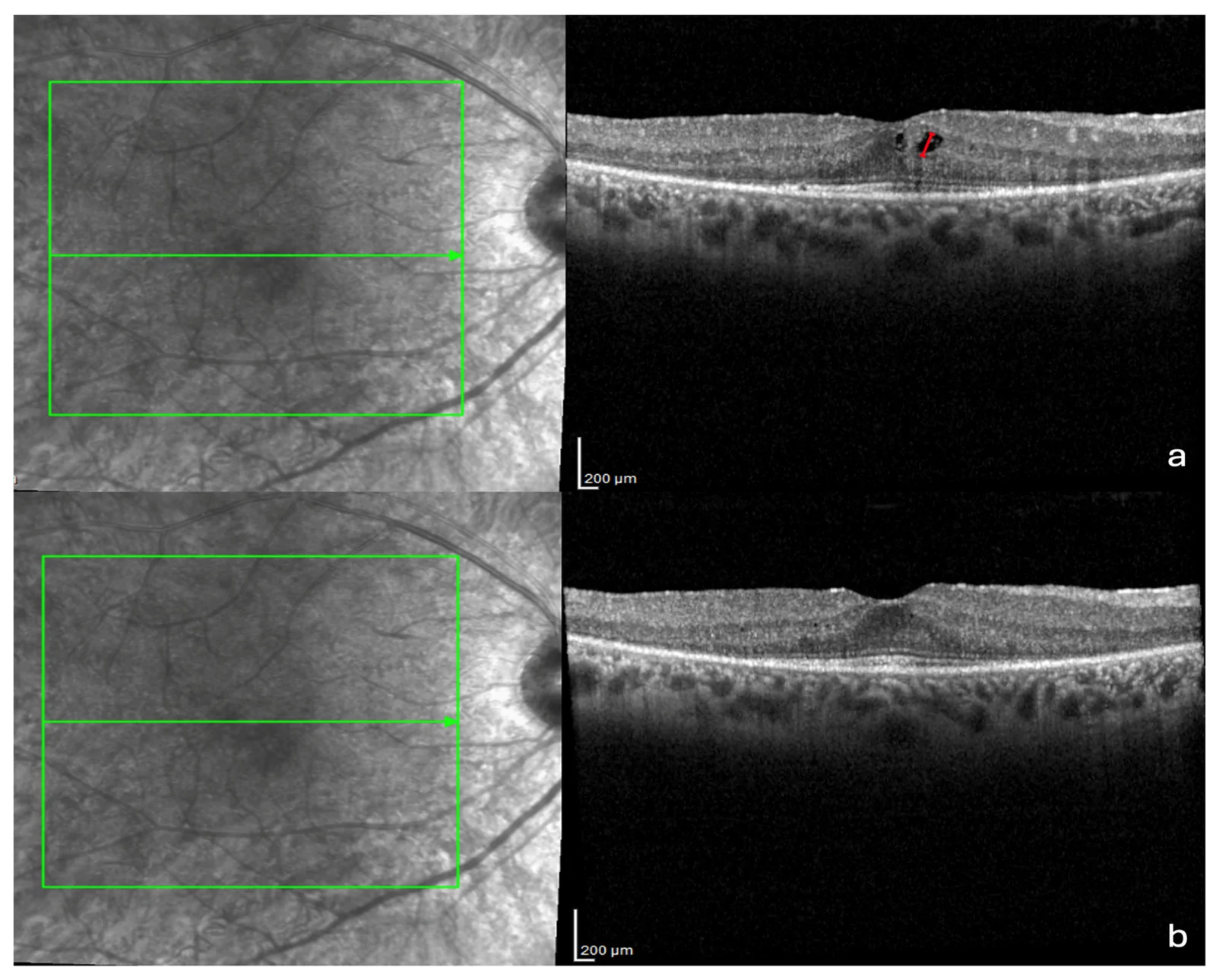
(**a**) Macular optical coherence tomography (OCT) features of microcystic oedema in a retinitis pigmentosa (RP) eye at baseline (V1). The green lines and arrows indicate the OCT scans on the en face retinal image that contribute to create a thickness map. The maximum diameter of the hyporeflective space of the cyst, indicated by the red calliper, was less than 30% of the total macular thickness (CMT = 276 microns). The best-corrected visual acuity (BCVA) was a Snellen fraction of 0.9. (**b**) Macular OCT scan after 12 months of intake of bromelain and curcumin (V2) in the same RP eye. We can see a marked reduction in the cystic spaces with a consequent reduction in CMT (CMT = 240 microns). The BCVA was a Snellen fraction of 1.0.

**Figure 2:**
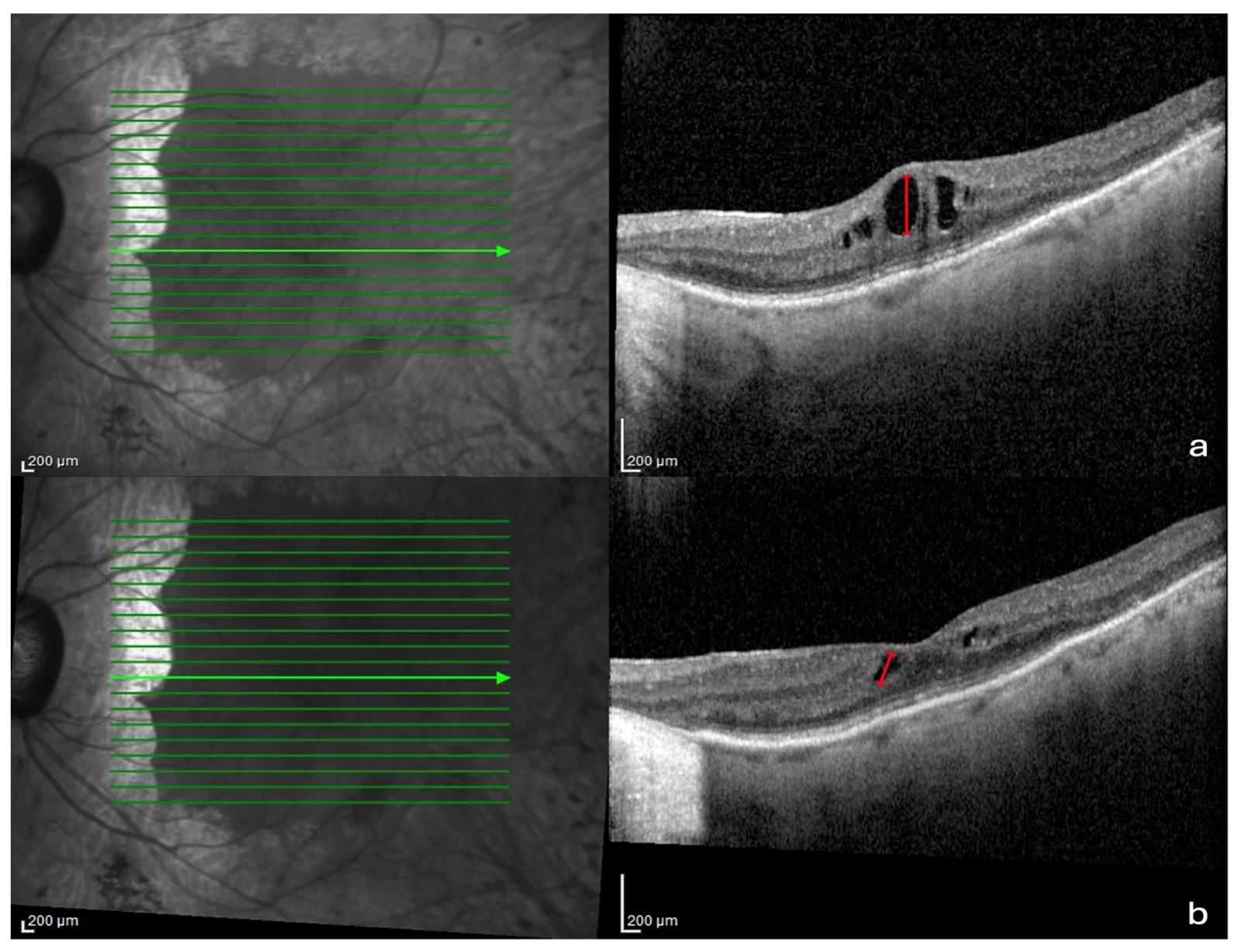
(**a**) Macular optical coherence tomography (OCT) features of macrocystic oedema in a retinitis pigmentosa (RP) eye at baseline (V1). The green lines and arrows indicate the OCT scans on the en face retinal image that contribute to create a thickness map. The maximum diameter of the hyporeflective space of the cyst, indicated by the red calliper, exceeded 30% of the total macular thickness (CMT = 364 microns). Best-corrected visual acuity (BCVA) was a Snellen fraction of 0.5. (**b**) Macular OCT scan after 12 months of intake of bromelain and curcumin (V2) in the same RP eye. It can be seen that there is a marked reduction in the cystic spaces and in CMT (CMT = 275 microns). The BCVA was a 0.6 Snellen fraction.

The documented CMT measurement through macular OCT allowed for the sub-grouping of 10 eyes with macrocystic CMO, in which the cystic component accounted for more than 30% of the total CMT, and 10 eyes with microcystic CMO, in which the cystic component accounted for less than 30% of the total CMT [21]. Representative cases of both microcystic and macrocystic IRD-related CMO at V1 (baseline) are shown in Figure 1a and Figure 2a, respectively.

As shown in Table 1, at V1 in the microcystic CMO group, the median best-corrected visual acuity (BCVA) was 0.95, with an interquartile range (IQR) of 0.77–1.00, and the median CMT was 280 microns (µm), with an IQR of 252–312 µm. At V2 (after 12 months of oral supplementation with bromelain and curcumin) in the same group, the median BCVA was 1.00 (IQR 0.67–1.00), and the median CMT was 277 µm (IQR 234–301 µm) (Table 1). In the microcystic group, although the median V2 BCVA was slightly improved with respect to V1, the difference in the same parameter between the two time points did not reach statistical significance (*p* = 0.665). Moreover, we detected a reduction in median V2 CMT as compared to V1; however, the difference in median CMT between V1 and V2 was also not statistically significant due to the wider distribution of the sample in the IQRs (*p* = 0.206) (Table 1).

On the other hand, in the macrocystic CMO group, the median BCVA at V1 was 0.60 (IQR 0.50–0.80), and the median BCVA at V2 was 0.60 (IQR 0.50–0.70). In the same group, the median CMT at V1 was 365 microns (IQR 343–372 µm), and at V2, the median CMT was 331 µm (IQR 311–350 µm). Unlike the microcystic-CMO group, in macrocystic CMO eyes, a statistically significant stability of visual function, measured by BCVA (*p* < 0.05), and a statistically significant reduction in CMT (*p* < 0.05) were observed, as detailed in Table 1.

Figure 2b shows a representative case of a reduction in macrocystic CMO at V2 in one RP patient whose BCVA changed from 0.4 at V1 to 0.6 at V2.

In Figure 1b and Figure 2b are two representative cases of relevant CMO reduction after 12 months of oral supplementation of bromelain and curcumin (V2) in two RP eyes, with micro- and macrocystic CMO, respectively.

As shown in Table 2, by inferential statistics, it was found that either median BCVA and CMT values were significantly different in the two groups at both time points (V1: BCVA microcystic versus macrocystic, *p* = 0.009; V2: BCVA microcystic versus macrocystic, *p* = 0.004; V1: CMT microcystic versus macrocystic, *p* < 0.001; V2: CMT microcystic versus macrocystic, *p* = 0.001).

## 3. Discussion

Our study aimed to retrospectively evaluate the effects of bromelain and curcumin supplementation in patients affected by inherited retinal dystrophies complicated by CMO who were refractory to standard treatments or reluctant to undergo such treatments chronically.

The examined IRD patient cohort consisted of individuals diagnosed with syndromic or non-syndromic RP. This condition is a degenerative disorder affecting the outer retina, leading to progressive deterioration and loss of photoreceptors with a predominantly centripetal pattern, resulting in a gradual decline in the peripheral visual field and, in advanced stages, impairment of central visual function. A variable percentage of cases (10–50%) ultimately develop CMO [2,6]. However, the presence of CMO is not a reliable predictor of visual acuity in RP, whereas CMT has been shown to exhibit a stronger association with the degree of visual acuity deterioration [22].

Syndromic or non-syndromic RP-related CMO has a multifactorial pathogenesis that can be broadly summarised into structural-degenerative and/or inflammatory-oxidative components, although tractional forms of CMO may occur [5,6]. Depending on the predominant pathogenic mechanism, the oedema may show a microcystic or macrocystic pattern. Generally, degenerative CMO are microcystic and are secondary to a cell loss mechanism [23], as happens in other neurodegenerative diseases like Multiple Sclerosis, Leber Optic Neuropathy, and Neuromyelitis Optica, in which the localization of the retinal cysts is mainly at the level of the inner nuclear layer (INL) [23,24]. Nevertheless, in some cases, CMO has a macrocystic pattern due to inflammatory-oxidative mechanisms determined mainly by BRB breakdown or retinal pigment epithelium (RPE) dysfunction [25]. In detail, BRB is made up of two components: the outer barrier, formed by tight junctions of RPE cells, and the inner barrier, constituted by tight junctions of vascular endothelial cells. Both these components may be damaged in RP-related degeneration. As a consequence, the toxic components released by degenerating retina, such as chemokines and cytokines, not blocked by the BRB filter, start and enhance the inflammatory cascade at the RPE level [25]. A dysfunctional RPE impairs the active transport of fluid from the subretinal space, which is essential for maintaining adhesion between the RPE and the photoreceptor layer [26]. Moreover, inflammation and degenerative oxidative stress have repercussions on Müller cells, causing redistribution of ion channels (particularly Kir4.1) and disruption of potassium and water homeostasis, which result in cellular swelling [27].

Recognising and addressing the macular complication is crucial, as the majority of these patients already exhibit severely compromised peripheral visual fields. Since eyes with RP show a globally reduced retinal thickness due to tissue degeneration, the value of ophthalmoscopic evaluation of these eyes alone does not allow for evaluating, in detail, the thinning of the outer macular layers. Conversely, OCT imaging allows us to adequately evaluate and quantify the CMT. This is crucial because chronic CMO [12] results in a profound decline in autonomy and overall worsening of quality of life.

Several treatments, both local and systemic, have been described in the literature for managing IRD-related CMO. Systemic administration of acetazolamide (AZM) is the therapeutic choice with the highest level of scientific evidence and is effective in many cases [14,28]; however, chronic usage as well as paediatric administration may affect renal function. In cases that are nonresponsive to AZM, intravitreal injections of anti-VEGF agents [14] or slow-release steroid implants [29] may be beneficial.

The therapeutic effects of AZM in RP-CMO have been suggested to have a dual action: decreasing retinal vascular leakage and augmenting active transport across the BRB [14,30]. Patients who responded to CAIs consistently showed combined drainage of fluids at the level of the INL and outer nuclear layer (ONL) [25]. The rationale for the usage of steroids in the management of RP-CMO is based on the identification of multiple T-cell subsets in vitreous samples [31] and inflammatory cells or proinflammatory cytokines in both aqueous and vitreous samples in RP patients [32]. Based on both CMT reduction and BCVA improvement (which are both outcomes of the present study), trials evaluating IVT-DEX versus CAIs (oral acetazolamide or topical dorzolamide) were conducted, and IVT-DEX demonstrated better efficacy [29]. However, the risks of steroid-induced intraocular pressure elevation and cataract formation must be considered in the case of chronic and recurrent therapy.

Due to the chronic nature of IRD-CMO, which is up to 50% of cases, involving those refractory to therapies as described by Arrigo et al. [10] in a longitudinal study conducted for 12 months, we selected the clinical records of those patients with inadequate response to the above-mentioned treatments or of those who were not eligible or not willing to undergo them due to reported adverse effects. For this reason, non-invasive, effective and chronically sustainable treatment of persistent IRD-CMO is an unmet need of such patients. For this reason, we retrospectively evaluated the effect of nutraceutical supplementation with bromelain and curcumin over a 12-month period in CMO-IRD-related patients who had not benefited from previous therapies administered for more than 6 months or who were unable to tolerate them.

We classified IRD-CMO as macrocystic or microcystic CMO and analysed two subgroups with sub-foveal EZ preservation detected by OCT scans. At baseline, the two cohorts showed different macular thicknesses and significantly different BCVAs, with better visual acuity in the degenerative microcystic group. Twelve months after the treatment, slightly improved BCVA and reduced CMT that did not reach statistical significance were found in the degenerative microcystic CMO. Conversely, in the vasogenic macrocystic group, although BCVA remained stable, CMT was reduced significantly. 

These observations may suggest that the anti-inflammatory and antioxidant effects of bromelain and curcumin are mainly exerted on the vasogenic compound of macrocystic CMO. However, the stability of VA and the reduction in the microcystic component in degenerative CMO, although not significant, indicate that this supplementation may have a role when other therapeutic strategies cannot be carried out.

It is known that curcumin, derived from the rhizome of Curcuma longa, has effects on human RPE (ARPE-19) cells in terms of protective properties against oxidative stress, inflammation, and the apoptotic process because of downregulation of the expression of genes involved in processes that lead to nuclear factor jB (NF-jB) activation [19,20]. Curcumin has been shown to reduce metabolic activity, leading to decreased cell proliferation without compromising cell survival. It can play its anti-inflammatory role even through the activation of transcriptional factors such as peroxisome proliferator-activated receptor-c (PPAR-c). In addition, it has a direct antioxidant property by inhibiting free radical production like H_2_O_2_ and provides significant protection against cell death [33,34].

On the other hand, bromelain has a direct effect on cytokine production. It can downregulate the secretion of IL-1β, IL-6 and TNF-α in activated immune cells when a condition of overproduction of cytokines has been promoted. Moreover, it can act even on the transcriptional side by reducing the expression of INF-γ and TNF-α [18].

Recent studies conducted in DMO have shown short- [20] and long-term [19] benefits only in terms of CMT reduction through either administering conventional therapies adjuvated by bromelain and diosmin or administering a combination of bromelain and curcumin, respectively. In these reports, BCVA did not show any statistically significant improvements, and bromelain was administered at a dosage of 500 mg/die.

A nutraceutical formula containing 160 mg of bromelain and 400 mg of curcumin was used in DMO, with a positive impact on both CMT and BCVA after 6 months of supplementation [35].

Based on these studies and on the results of the present series, we believe that these natural compounds may favour, in the long term, a reduction in persistent IRD-CMO, especially in cases with macrocystic oedema. Furthermore, at least in eyes with preserved integrity of the sub-foveal EZ, these compounds may contribute to avoiding BCVA changes by counteracting the inflammatory process that threatens foveal photoreceptors.

We acknowledge the main limitations of the present study, which are the small sample size and the lack of a control group. The stringent inclusion criteria and the retrospective study design limited the opportunity to recruit larger IRD-CMO cohorts. We could not identify a control group, meaning IRD-CMO eyes left untreated, due to ethical principles of ophthalmological care.

Further prospective studies are needed to assess the role of the nutraceutical supplementation as a therapeutic option in naïve and/or refractory IRD-CMO cases.

## 4. Materials and Methods

### 4.1. Study Design and Participants

A cohort of approximately 70 patients, followed up from 2015 to 2024 by the Clinical and Research Center of Neurophthalmology and Rare Disease Unit of IRCCS-Fondazione Bietti, with chronic IRD-related CMO, refractory to conventional therapies, including topical or systemic acetazolamide (*n* = 55), as well as intravitreal injections of slow-release dexamethasone implants (*n* = 10) or anti-VEGF agents (*n* = 5), was identified through the evaluation of patients’ clinical records.

From this cohort of patients, we selected the study population of the present retrospective study through the below-mentioned inclusion and exclusion criteria.

### 4.2. Inclusion Criteria Were

- Age between 18 and 60.
- Clinically assessed IRD condition with conclusive genetic testing.
- Presence of persistent IRD-related CMO for more than 6 months after standard therapy administration (systemic or topical acetazolamide, repeated intravitreal slow-release dexamethasone implants or repeated intravitreal administration of anti-VEGF).
- Patient with IRD-related CMO reluctant to continue systemic or topical acetazolamide, repeated intravitreal slow-release dexamethasone implants, or repeated intravitreal administration of anti-VEGF.
- Patient with IRD-related CMO with continuous 12-month intake of curcumin and bromelain tablets, Retinil Forte^®^, Mesofarma S.R.L., Mosorrofa, Italy (1 capsule every day on fast, containing 250 mg of bromelain and 100 mg of curcumin), after suspension of the above-mentioned CMO therapeutic options because they were ineffective or not tolerated.
- Patient with available clinical records of BCVA, ophthalmological evaluation, fundus imaging and OCT scans at the time of initiation of intake of bromelain and curcumin tablets and after 12 months of intake.
- Eyes with preserved sub-foveal EZ at V1 and V2 observed by OCT scans.

### 4.3. Exclusion Criteria Were

- Ocular surgery 6 months before the initiation of the intake of bromelain and curcumin tablets;
- CMO due to diabetic retinopathy, retinal vascular occlusions or other systemic illnesses;
- Presence of macular traction (epiretinal membrane, vitreomacular adhesion, vitreomacular traction, lamellar or full-thickness macular hole) on OCT scans;
- CMO due to ocular trauma;
- CMO due to inflammatory systemic, retinal and/or choroidal disorders.

By considering all these inclusion and exclusion criteria, we collected data from 20 eyes.

After contacting the patients and explaining the purpose of data collection for this study, each subject was invited to sign an informed consent form. All procedures performed in this study adhered to the tenets of the Declaration of Helsinki. The local Ethical Committee (Comitato Etico Territoriale Lazio Area 5, IRCCS Istituti Fisioterapici Ospitalieri, Roma, Italy) approved the study protocol (N. 408/FB/25) on 21 October 2025.

For each patient, two visits were considered: the baseline visit (V1), during which persistent CMO was observed and bromelain and curcumin intake was initiated by the patient, and the follow-up visit (V2), 12 months after beginning bromelain and curcumin intake.

### 4.4. Ophthalmological and Visual Assessments

We collected, for each eye enrolled in the study, data about a comprehensive ophthalmological examination, including slit-lamp evaluation of the anterior and posterior segments, the intraocular pressure measurement by applanation tonometry (Applanation Goldman Tonometer, Inami, Tokio, Japan), the BCVA measurement at 4 m in Snellen fraction, and a near vision measurement tested by Jaeger Reading Chart at 40 cm. 

We also recorded data on a dilated fundus examination with a +90D non-contact lens (Volk Optical, Mentor, OH, USA).

### 4.5. Imaging and Structural Assessments

Data from a structural assessment of the macula executed by ultrawide-field fundus photographs by (Optos California PLC, Dunfermline, UK) fundus autofluorescence (FAF) imaging at 55° and optical coherence tomography (OCT) performed with Heidelberg Spectralis OCT (HRA + OCT, Heidelberg Engineering, Heidelberg, Germany) were collected for each patient enrolled at V1 and V2. A standardised OCT protocol was employed, consisting of 25 B-scans arranged within a 20° × 20° raster pattern centred on the fovea, with an interscan distance of 258 µm. Image acquisition was performed using the Automatic Real-Time (ART) mode, and follow-up examinations were aligned using the built-in progression algorithm of the device. Near-infrared reflectance images were captured simultaneously with a default scanning angle of 30°.

The OCT image quality strength index of the acquired scan was >25. Scans that did not fulfil the above-mentioned criteria were excluded from the analysis.

Quality control and APOSTEL 2.0 recommendations according to the published criteria were followed [36].

For the latter exams, we collected only the data of the CMT from OCT scans defined by automatic built-in segmentation; in cases of failed segmentation, each scan was carefully reviewed by two expert graders (M.D.A. and L.B.).

Based on the OCT data of enrolled patients, which displayed a generally reduced thickness due to the degenerative nature of IRD, we identified two different subgroups of IRD-related CMO:

Microcystic CMO, in which the cystic component accounted for less than 30% of the CMT.

Macrocystic CMO, in which the cystic component accounted for more than 30% of the CMT. This classification has already been used in a different cohort of patients affected by a different retinal disease, reported in an amplified version by Helmy et al. [37].

During data selection at baseline, when both eyes of the same patient exhibited oedema, with distinct morphological characteristics in either eye that warranted classification into different study groups (i.e., microcystic and macrocystic CMO), each eye was considered to be an independent observational unit and analysed separately. We ensured that the CMO behaviour (either microcystic or macrocystic) was such 6 months before study inclusion and throughout the study’s duration.

### 4.6. Statistical Analysis

BCVA and CMT were summarised using median and interquartile range values at each sampling time point for both groups (micro- and macrocystic oedema).

Each parameter’s sampling time points at the baseline and after the treatment were compared using Friedman tests for both groups.

Lastly, BCVA and CMT of the micro- and macrocystic oedema groups were compared by means of the Mann–Whitney test at each sampling time point.

A *p*-value of 0.05 was considered as significant for the Friedman tests, while it was set to 0.025 for the Mann–Whitney tests to compensate for the number of multiple comparisons using the Bonferroni method (0.05/2 = 0.025).

## 5. Conclusions

Oral bromelain and curcumin supplementation showed a significant anatomical improvement and functional stabilisation in macrocystic CMO eyes, whereas microcystic CMO remained structurally and functionally stable over a 12-month period.

Our findings support the hypothesis that IRD-related macrocystic CMO, characterised by a stronger inflammatory–vasogenic component than degenerative microcystic CMO, is more responsive to nutraceutical molecules with anti-inflammatory and antioxidant properties.

Given the long-term usage limitations of carbonic anhydrase inhibitors and intravitreal therapies in IRD-related CMO eyes, supplementation with nutraceutical molecules may represent a safe and alternative strategy in refractory macrocystic CMO.

## Figures and Tables

**Table 1:** Descriptive and inferential statistics (Friedman test) between V1 and V2 time points of median values of best-corrected visual acuity and central macular thickness in eyes with cystoid macular oedema related to inherited retinal dystrophy.

	*N* ^a^		V1 ^b^	V2 ^c^	V1 ^b^ vs. V2 ^c^ (S; *p*)
Microcystic CMO ^d^	10	BCVA ^e^	0.95; (0.77–1.00)	1.00; (0.67–1.00)	0.20; 0.655
		CMT ^f^	280; (252–312)	277; (234–301)	1.60; 0.206
Macrocystic CMO ^d^	10	BCVA ^e^	0.60; (0.50–0.80)	0.60; (0.50–0.70)	4.00; 0.046
		CMT ^f^	365; (343.0–372)	331.0; (311.0–350.0)	3.60; 0.035

^a^ *N* = Number of eyes; ^b^ V1 = baseline time point; ^c^ V2 = assessment after 12 months of bromelain and curcumin intake; ^d^ CMO = cystoid macular oedema; ^e^ BCVA = best-corrected visual acuity measured in Snellen fraction; ^f^ CMT = central macular thickness measured in microns. *Statistical significance set at p < 0.05*.

**Table 2:** Inferential statistics (Mann–Whitney test) between data of micro- and macrocystic oedema at V1 and V2 time points.

	V1 ^a^ (W, *p*)	V2 ^b^ (W, *p*)
BCVA ^c^	147.5; 0.009	142.0; 0.004
CMT ^d^	55.0; <0.001	64.0; 0.001

^a^ V1 = baseline time point; ^b^ V2 = assessment after 12 months of bromelain and curcumin intake; ^c^ BCVA = best-corrected visual acuity measured in Snellen fraction; ^d^ CMT = central macular thickness measured in microns. *Statistical significance set at p < 0.025*.

## Data Availability

The original contributions presented in this study are included in the article. Further inquiries can be directed to the corresponding author.

## Abbreviations

The following abbreviations are used in this manuscript:

ART	Automatic Real-Time
AZM	Acetazolamide
BCVA	Best-Corrected Visual Acuity
BRB	Blood–Retinal Barrier
CAIs	Carbonic Anhydrase Inhibitors
CMO	Cystoid Macular Oedema
CMT	Central Macular Thickness
DMO	Diabetic Macular Oedema
EZ	Ellipsoid Zone
FAF	Fundus Autofluorescence
INL	Inner Nuclear Layer
IOP	Intraocular Pressure
IQR	Interquartile Range
IRD	Inherited Retinal Dystrophies
IVT-DEX	Slow-Release Intravitreal Dexamethasone Implant
LE	Left Eye
N	Number of Eyes
NF-jB	Nuclear Factor jB
OCT	Optical Coherence Tomography
ONL	Outer Nuclear Layer
PPAR- c	Peroxisome Proliferator-Activated Receptor-c
RD	Retinitis Pigmentosa
RE	Right Eye
RPE	Retinal Pigment Epithelium
SD	Standard Deviation
TNF-α	Tumour Necrosis Factor α
VEGF	Vascular Endothelial Growth Factor
